# Efficacy of a Novel Dual-Layer Plastic Stents for Malignant Biliary Obstruction

**DOI:** 10.3390/jcm14030764

**Published:** 2025-01-24

**Authors:** Masanari Sekine, Masashi Ijima, Satoaki Noguchi, Eishin Kurihara, Tsutomu Kobatake, Taku Mizutani, Ryo Hashimoto, Kayoko Aoyama, Goya Sasaki, Azumi Sato, Shu Kojima, Hirosato Mashima

**Affiliations:** 1 Department of Gastroenterology, Jichi Medical University Saitama Medical Center, Saitama 330-8503, Japan; mizutani.taku@jichi.ac.jp (T.M.); hashimoto.ryo@jichi.ac.jp (R.H.); kayoko.aoyama@jichi.ac.jp (K.A.); sasaki.58@jichi.ac.jp (G.S.); n23045@jichi.ac.jp (A.S.); akitajounosuke@gmail.com (S.K.); hmashima@jichi.ac.jp (H.M.); 2 Department of Gastroenterology, Ota Memorial Hospital, Gunma 373-8585, Japan; ijima0711@gmail.com (M.I.); n.satoaki0616@gmail.com (S.N.); eishin.kurihara.dec.18@gmail.com (E.K.); t.kobatake.05267@ota-hosp.or.jp (T.K.)

**Keywords:** plastic stent, malignant biliary obstruction, polytetrafluoroethylene (PTFE)

## Abstract

**Objectives:** In hepatopancreatic diseases, stenting is widely employed to manage cholangitis and obstructive jaundice. Stent materials are primarily categorized as plastic or metal. Plastic stents have notable advantages, such as reduced likelihood of peripheral bile duct obstruction, a lower cost, and the ease of replacement compared to metallic stents. However, their patency period is shorter due to narrower diameters. Plastic stents are typically composed of materials like polyurethane or polyethylene. To improve patency, new dual-layer stents combine polyurethane with polytetrafluoroethylene (PTFE). PTFE, used in the inner layer, is expected to prevent biofilm formation. This study aimed to assess the clinical efficacy of this dual-layer stent. **Methods:** A retrospective analysis was performed on 48 cases (Group R) using REGULUS^®^ from November 2022 to November 2023 and 30 cases (Group IS) using inside-type plastic stents from January 2020 to November 2023 for malignant hilar and intrahepatic bile duct obstructions. Stent patency and clinical outcomes were compared between the groups. **Results:** There was no significant difference in the recurrent biliary obstruction (RBO) rate between the groups (*p* = 0.644). The time to recurrent biliary obstruction (TRBO) was 74 days in Group R and 118 days in Group IS, with no significant difference (*p* = 0.219). **Conclusions:** The dual-layer stent placed across the papilla demonstrated comparable clinical outcomes to inside-type stents. The PTFE inner layer likely reduces biofilm formation, enhancing patency. Across-the-papilla placement may facilitate reinterventions in challenging cases, broadening stent options.

## 1. Introduction

Stents are commonly used for drainage in cases of cholangitis or obstructive jaundice in the treatment of biliopancreatic diseases. In particular, in cholangiocarcinoma of the hilar region, various factors influence the choice of biliary drainage method [1], including the localization and extent of the tumor, surgical resectability, estimated remnant liver volume, relationship with arterial structures, presence of preoperative treatments such as portal vein embolization, and predicted prognosis. Endoscopic nasobiliary drainage (ENBD) is recommended for preoperative drainage because of its lower incidence of cholangitis [2]. However, for cases in which prolonged preoperative waiting periods are anticipated, such as in patients undergoing preoperative therapies, ENBD can negatively impact the quality of life (QOL) [3]. In such situations, a plastic stent (PS) is often used as an initial strategy.

For unresectable cases, the 3rd edition of the Guidelines for the Treatment of Biliary Tract Cancer [4] recommends either an uncovered self-expanding metal stent (UC-SEMS) or plastic stent. Stents are typically composed of plastic or metal. Plastic stents offer several advantages over metal stents, including a reduced risk of occlusion of peripheral bile duct branches, ease of replacement, and cost-effectiveness. However, their smaller diameter has led to a shorter patency period [5]. Biofilm formation is a known contributor to the occlusion of these stents [6], prompting the exploration of various strategies, such as silver coatings, to prevent this complication. Recently, the effectiveness of inside-type plastic stents has been reevaluated [7,8,9]. The UC-SEMS outperforms the PS in terms of patency duration, making it the preferred choice. However, as multimodal treatment advances and the patient prognosis improves, the limitations of UC-SEMS, particularly its irreversibility, become more apparent.

Re-intervention in UC-SEMS cases is challenging because of the need to navigate through the mesh structure of the stent. Considering a long-term ERCP strategy beyond the initial patency period, a more comprehensive approach is necessary. Recently, the IS has been commercialized and gained attention. Traditional PS placement, which extends into the duodenum, may lead to biofilm formation from duodenal flora, causing stent occlusion. In contrast, IS placement confines the stent within the bile duct, preserving the function of the sphincter of Oddi and potentially extending patency compared to conventional PSs. However, in some cases, especially when considering re-intervention, across-the-papilla stents may be more beneficial.

**Figure 1:**
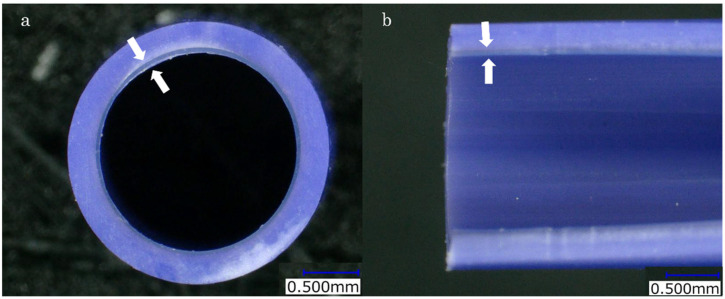
Structure of the dual-layer stent. The layer between the white arrows is the inner PTFE layer. (**a**) Enlarged view of the cross-sectional slice. (**b**) Enlarged view of the longitudinal sectional slice.

This study aimed to assess the effectiveness of a novel dual-layer stent composed of polyurethane with an inner polytetrafluoroethylene (PTFE) layer (Figure 1) to minimize biofilm adhesion and potentially extend stent patency.

## 2. Methods Study Design and Patients

Transpapillary stent placement was performed in 78 patients with malignant biliary obstruction at 2 medical centers in Japan: Jichi Medical University Saitama Medical Center and Ota Memorial Hospital. All the patients had malignant tumors. In addition, all cases involved obstructions of the hilar or intrahepatic bile ducts. We retrospectively compared two groups: 48 cases (R group) in which REGULUS^®^ was used between November 2022 and November 2023 and 30 cases (IS group) in which inside-type plastic stents were used between January 2020 and November 2023. These cases involved malignant tumors that caused obstruction of the hilar and intrahepatic bile ducts. The patency periods and safety profiles were retrospectively analyzed.

### 2.1. Inside Stents (ISs)

Before commercial products became available, in the first report, ISs were handmade by attaching nylon threads to conventional stents [10]. This stent is made only of polyethylene. This product is available in two types based on flexibility: a deep-angle type and a light-angle type, allowing for selection tailored to the anatomy of the targeted bile duct. In addition, the stent is available in the diameters of 7 and 8.5 Fr. The larger diameter option is suitable for cases in which longer stent patency is desired. In addition to extended patency periods, ISs offer several practical advantages. For instance, it is possible to place up to three stents without undue stress on the papilla. ISs are also beneficial in cases of long biliary strictures, where the length of the conventional transpapillary stents may be insufficient. In all cases, the ISs were placed above the papilla. “Above the papilla” refers to a method where the proximal end of the stent is placed within the bile duct, without extending it into the duodenum.

### 2.2. The Novel Dual-Layer Plastic Stent

The novel stent has an outer layer made of polyurethane with high resilience, which softens at body temperature (approximately 37 °C), and an inner layer composed of PTFE, which offers excellent lubricity. The lumen was designed to be relatively wide, ensuring a high degree of fit within the bile duct while maintaining superior patency. The stent is available in two diameters, 7 Fr and 8.5 Fr, and has five lengths: 5, 7, 10, 12, and 15 cm. The stents are designed with pre-curves and are equipped with flaps, which reduces the risk of dislodgement. In all cases, the novel stent was placed across the papilla. “Across the papilla” refers to a method where the proximal end of stent extends into the duodenum for placement.

### 2.3. Procedure

Experienced endoscopists at two medical centers performed all endoscopic procedures. Multidetector computed tomography (CT) or magnetic resonance cholangiopancreatography (MRCP) was used to plan stent placement for the bile duct. A standard duodenoscope (JF-260V, TJF-260V, or TJF-290; Olympus Medical Systems, Tokyo, Japan) was used. After the cannulation of the bile duct, a guidewire was passed through the obstructed tumor. Endoscopic sphincterotomy (EST) was performed in all cases, and a stent was placed in the intrahepatic bile duct. The operator determined the type, number, size, and length of stents. REGULUS^®^ was placed across the papilla, while inside-type plastic stents were placed above the papilla.

Two patterns of development after the placement of stents were observed: (1) Stent replacement due to the occurrence of stent occlusion with acute cholangitis and (2) stent replacement without stent occlusion. Strategic replacement without stent occlusion was excluded at the time of analysis. The acute cholangitis rate was analyzed using the Kaplan–Meier method. The stent occlusion rate was defined as the proportion of stent occlusion at 10 days, 20 days, 30 days, 60 days, and 90 days after stent placement and was analyzed using the Kaplan–Meier method.

### 2.4. Statistical Analyses

Statistical analyses were performed using the EZR software program (ver.1.68) [11], employing Fisher’s exact test, the Wilcoxon signed-rank test, and the log-rank test to compare time to recurrent biliary obstruction (TRBO). For adverse events such as cholangitis related to stents, RBO, and TRBO, we referred to the Tokyo Criteria 2024. When analyzing stent patency using the Kaplan–Meier method, patients without stent occlusion and cholangitis were excluded at the time of analysis.

#### Ethical Aspects and Consent to Participate

This study was approved by the ethics committee of Saitama Medical Center, Jichi Medical University, and conducted in accordance with the Declaration of Helsinki. We obtained written informed consent or had the right to refuse information disclosure and the opt-out opinion from all patients.

## 3. Results

Patient backgrounds are shown in Table 1. There were no significant differences in sex or age between groups. The proportion of obstructions caused by bile duct cancer was higher in the R group (89.6%) than in the IS group (73.3%) (*p* = 0.0338). In addition, the IS group had a higher prevalence of HCC and liver metastases, with a greater proportion of intrahepatic bile duct obstruction compared to the R group.

The study outcomes are presented in Table 2. All patients in both groups underwent EST. In the IS group, 12 cm-long stents were predominantly used, while in the R group, stents with a length of 10 to 15 cm were mainly used. The R group frequently had two stents placed, followed by three, whereas the IS group typically had one stent placed, followed by two stents (*p* = 0.00208). This is likely because the IS group had a higher rate of obstruction limited to the intrahepatic bile duct, such as those caused by liver metastases, whereas the R group had more cases of bile duct obstruction in the hilar region.

**Figure 2:**
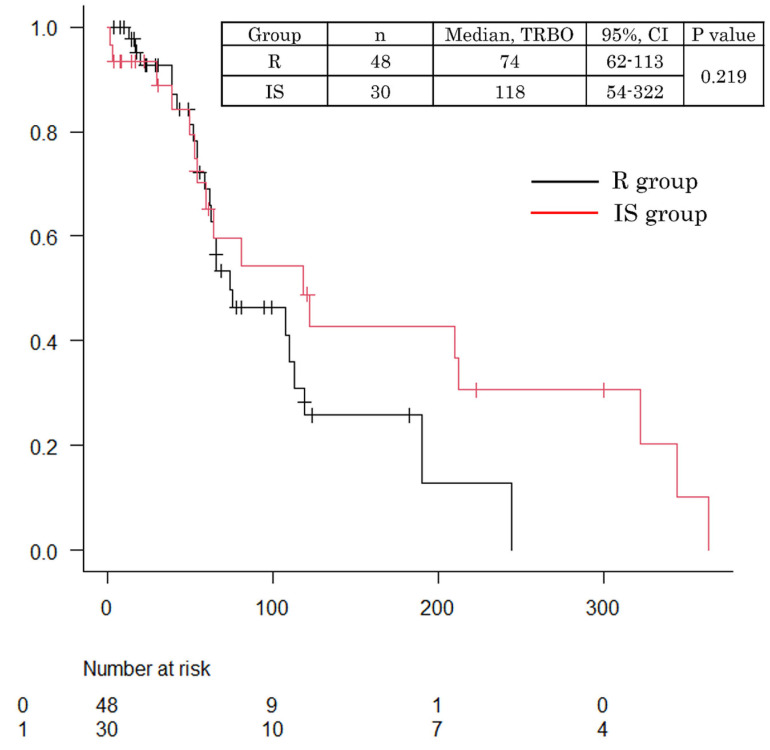
Kaplan–Meier analysis of time to recurrent biliary obstruction (RBO). There was no statistically significant difference in the median time to RBO (TRBO) between the R group and the IS group (74 days vs. 118 days, 95% confidence interval [CI], *p* = 0.219).

**Figure 3:**
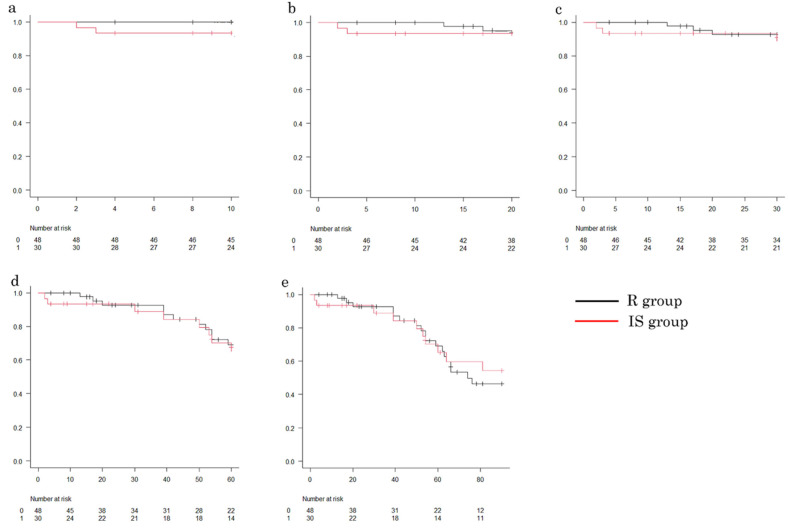
Kaplan–Meier analysis of TRBO. (**a**) There was no statistically significant difference in the median TRBO up to day 10 between the R group and the IS group (*p* = 0.0712). (**b**) There was no statistically significant difference in the median TRBO up to day 20 between the R group and the IS group (*p* = 0.89). (**c**) There was no statistically significant difference in the median TRBO up to day 30 between the R group and the IS group (*p* = 0.517). (**d**) There was no statistically significant difference in the median TRBO up to day 60 between the R group and the IS group (*p* = 0.692). (**e**) There was no statistically significant difference in the median TRBO up to day 90 between the R group and the IS group (*p* = 0.78).

There was no significant difference in the rate of acute cholangitis between the groups (*p* = 0.644). The median TRBO was 74 and 118 days in the R and IS groups, respectively, with no statistically significant differences (*p* = 0.219, Figure 2). Upon detailed analysis, the R group demonstrated longer patency than the IS group up to day 10, although the difference was not statistically significant (*p* =0.0712, Figure 3a). Around day 20, the patency curves of the R and IS groups began to converge (*p* =0.89, Figure 3b). By days 30 and 60, the curves almost entirely overlapped (*p* =0.517, 0.692, Figure 3c,d). Around day 90, the IS group demonstrated a slightly longer patency curve than the R group, although this difference was not statistically significant (*p* =0.78, Figure 3e).

Adverse events included one case of hyperamylasemia in the R group and three cases of hyperamylasemia and one case of cholangitis in the IS group, with no significant difference between the groups (Table 2).

## 4. Discussion

Bile duct cancer is more likely to cause obstructive jaundice than pancreatic cancer, and hilar bile duct cancer almost always leads to obstruction. Consequently, stent placement is often required in patients with unresectable tumors. Stents are primarily divided into plastic and metal types, each with advantages and disadvantages. Metal stents, known for their superior patency, have historically been preferred with stent-in-stent and side-by-side placement [12]. Previous reports, including randomized control trials (RCTs), have indicated that UC-SEMSs outperform plastic stents in terms of patency, making UC-SEMSs the recommended choice. However, advancements in chemotherapy have extended patient survival [13,14], and the disadvantages of non-removable UC-SEMS have become more apparent. This is particularly evident during re-interventions following stent dysfunction, as the mesh holes of the UC-SEMS require complex navigation, which significantly complicates the procedure.

As a result, there is an increasing need to consider long-term stent selection strategies, necessitating even longer stent patency periods or reintervention that account for re-interventions, rather than focusing solely on the initial patency period.

ISs have recently been introduced commercially and have shown promise. The IS is placed entirely within the bile duct without extending into the duodenal lumen, preserving the function of the sphincter of Oddi. Inside-type plastic stents have been reported to be effective for malignant strictures in hilar and intrahepatic bile ducts [7,8,9]. The benefits of plastic stents include the potential for longer patency with inside-type stents. Nonetheless, across-the-papilla placement may be preferable in scenarios where ease of replacement and reintervention are anticipated. Retrospective studies have reported that the IS offers longer stent patency than the PS, extending into the duodenal lumen. Kurita et al. conducted a prospective RCT demonstrating that ISs significantly prolonged patency compared with plastic stents in cases of unresectable malignant hilar strictures [15]. Similarly, a prospective study by Kogure et al. reported a 100% success rate for IS placement and removal, indicating that handling ISs pose no significant challenges [16]. When comparing ISs with UC-SEMS, retrospective studies have yielded mixed results, with no definitive conclusions. However, a recent prospective RCT by Kanno et al. found no statistically significant difference in the TRBO between ISs (250 days) and UC-SEMSs (361 days) in cases of unresectable malignant hilar biliary strictures [7]. Based on these findings, ISs, which can be removed, may be the preferred first-line treatment for such conditions.

For preoperative cases, no significant differences in stent dysfunction rates or adverse events were observed between the ENBD, the plastic stent extending into the duodenum, and IS groups [17]. In addition, Ishiwatari et al. reported no marked differences in the rates of cholangitis or adverse events between the plastic stent and IS groups [18].

The widely accepted theory regarding stent occlusion is that both bacterial biofilms and biliary sludge play central roles [6]. The biliary sludge associated with stent occlusion differs from cholesterol-rich sludge commonly linked to gallstone formation. Instead, this sludge is primarily composed of bilirubin calcium and calcium palmitate crystals, which are formed by bacterial enzymes. The formation of a biofilm, driven by the adhesion of various proteins not naturally present in bile, such as fibronectin, vitronectin, laminin, fibrin, and collagen, on the inner surface of the stent, is known to play a significant role in initiating sludge accumulation. The most critical factor in this process is bacterial colonization. Among aerobic bacteria, Gram-positive *Enterococcus* species and Gram-negative *Escherichia coli* and *Klebsiella* species are most commonly observed, while anaerobic bacteria such as *Clostridium* species are also implicated. The synergistic effect of bacterial adhesion and biofilm formation triggered by these bacteria is known to be a key factor in stent occlusion. Biofilms typically do not become thick enough to cause complete stent occlusion. Longitudinal evaluations of removed biliary plastic stents have shown that the inner layer of the biofilm is less than 0.5 mm thick, and occlusion is primarily caused by debris, sludge, and food particles. Scanning electron microscopy (SEM) observations of stents reveal that biofilm formation begins on the stent’s inner surface approximately four weeks after placement. Over time, the biofilm thickens relatively uniformly, and its surface becomes firmer. By about eight weeks after placement, the biofilm becomes covered with sludge, rapidly narrowing the stent’s inner diameter. This suggests that while the biofilm is involved in the initiation of stent occlusion, the thickening of the biofilm itself is not the direct cause of complete blockage. Instead, the biofilm may make the inner surface of the stent irregular, facilitating the accumulation of sludge and debris. Currently, no method has been identified to completely inhibit biofilm formation.

In this context, a dual-layer stent with an outer layer composed of polyurethane and an inner layer of PTFE was released. The ability of PTFE to prevent biofilm adhesion has been reported previously [19]. In addition to the expected patency benefits of PTFE, the use of polyurethane for the outer layer is anticipated to improve the fit of the stent within the bile duct.

In the present study, the dual-layer plastic stent showed similar results to the inside-type stent. Although there was no significant difference in the TRBO between the R and IS groups, the median TRBO was longer in the IS group at 118 days. Typically, across-the-papilla stents are associated with shorter patency periods than those placed above the papilla. The PTFE inner layer in our dual-layer stent likely plays a role in reducing biofilm formation. Consequently, although the stent was placed across the papilla, it achieved a patency period similar to that of an above-the-papilla stent. Notably, both groups maintained a patency rate >70% at 64 days. Although no significant differences were observed, the dual-layer plastic stent tended to have a longer patency period than the IS within the first 10 days and demonstrated a higher patency rate up to 20 days. Between days 30 and 60, both stents showed almost equivalent results. This is believed to be owing to the effects of PTFE. The high lubricity of PTFE likely prevents the early adhesion and retention of residual food and bile sludge while also inhibiting biofilm formation. As a result, the dual-layer plastic stent achieved a higher patency rate in the early phase and maintained a patency rate comparable to that of the IS in the later phase.

However, after approximately 70 days, a difference began to emerge, possibly owing to damage to the PTFE layer from the digestive fluids, leading to biofilm formation. This may indicate a limitation of PTFE, which emphasizes the need for continued innovation in stent materials. Nonetheless, when across-the-papilla stents can achieve longer patency periods than previous stents, straight-type plastic stents may facilitate stent removal and replacement via a guidewire inserted through the stent’s opening on the duodenal side.

ISs are designed with two types of pre-curves to match the angle of the bile duct and are equipped with flaps, which reduce the risk of dislodgement. No dislodgements were observed in the present study. REGULUS^®^ features a polyurethane outer layer, which softens at body temperature (approximately 37 °C), reducing axial force and minimizing stress on the intrahepatic bile ducts when placed across the papilla. However, its excessive softness raises concerns regarding early occlusion and dislodgement. In this study, the thin PTFE inner layer may have contributed to maintaining optimal stiffness, supporting prolonged patency.

A subset of cases presents challenges in achieving selective bile duct cannulation during the initial ERCP. These cases may also involve prolonged or complicated stent exchange. The primary cause is severe tumor-induced strictures, which can make guidewire placement difficult, even after stent removal. In some instances, selective cannulation is impossible. In such cases, transpapillary plastic stents are preferred. During subsequent exchanges, a guidewire was advanced through the existing transpapillary plastic stent. The stent was then removed using a snare or a similar tool, and a new stent was placed. This approach ensures greater reliability and reduces the procedure time compared to stent removal and replacement without a pre-placed guidewire. Given these advantages, transpapillary plastic stents are actively chosen over ISs in cases where selective bile duct cannulation is expected to be difficult.

In vitro studies [19] have demonstrated that Teflon stents with low friction coefficients and stents coated with hydrophilic materials can effectively inhibit bacterial colonization and sludge formation. Because bacterial adhesion to plastic stents is closely linked to surface hydrophobicity, hydrophilic coatings have been actively researched. Although initial studies reported promising results, subsequent prospective large-scale studies failed to demonstrate a significant extension of the stent patency periods. The potential reasons for this include (1) damage to the coating surface caused by guidewire manipulation during stent placement or duodenal bile reflux and (2) gradual degradation of the hydrophilic coating over time before stent occlusion occurs.

With regard to PTFE, an in vitro study by Coene et al. [19] revealed that Teflon exhibited the lowest friction coefficient compared to commonly used materials such as polyurethane and polyethylene. However, the inherent hardness and rigidity of Teflon pose the risk of biliary tract injury or duodenal perforation, rendering it less suitable as a practical material. To address these limitations, a novel two-layer plastic stent was developed that integrates the advantages of polyurethane, which is known for softening in vivo, with PTFE. This design aims to overcome these drawbacks while preserving the benefits of both materials.

A notable finding from this study pertains to the presence of side holes. In straight stents without side holes, the friction coefficient of the stent material is a key determinant of the deposition rate. The addition of side holes improved stent patency but significantly influenced the deposition rates. Interestingly, the superior performance of Teflon was counteracted by microfluidic turbulence caused by inner-wall irregularities or the presence of side holes. Thus, the differences in material performance were effectively neutralized by the presence of side holes. Conversely, the absence of side holes increases the risk of occlusion at both the bile duct and duodenal ends, likely due to contact with the ductal or duodenal walls. Consequently, the researchers emphasized that, if side holes are necessary in plastic stents, structural modifications to minimize inner-wall irregularities are critical.

PTFE offers remarkable versatility in terms of its physical properties, with thicknesses ranging from a few microns to several centimeters. This allows PTFE to be tailored to satisfy diverse operational environments and performance requirements. For instance, thin PTFE layers are ideal for applications requiring flexibility and non-stick properties, whereas thicker PTFE layers provide enhanced structural strength and abrasion resistance. Thin layers offer increased flexibility and conformability, whereas thicker layers are more rigid and are suitable for high-load and high-pressure applications.

The two-layer design of the novel stent is structurally sound and innovative. Nonetheless, future developmental challenges include optimizing the thickness ratio of the outer polyurethane layer to the inner PTFE layer, evaluating the necessity and positioning of the side holes from a fluid dynamics perspective, and determining their optimal quantity and placement. In addition, variations in stent shape, such as straight or pigtail configurations, require further investigation.

By maintaining the bacterial colonization and sludge inhibition properties of the PTFE inner layer while optimizing the stent design, including shape and side hole configurations, further improvements in the stent patency duration are anticipated.

The limitations of this study include its single-center retrospective design and small sample size. Further studies with larger sample sizes are required to provide stronger evidence.

## 5. Conclusions

Hilar cholangiocarcinoma requires a wide range of drainage strategies owing to anatomical variations in bile duct branching and the extent of tumor spread. The same applies to malignant tumors that affect intrahepatic bile ducts or hilar regions. Our findings demonstrate that the new dual-layer plastic stent offers patency rates comparable to those of ISs, which have been reported to achieve patency equivalent to SEMSs.

Including this new dual-layer plastic stent, alongside conventional options such as stent-in-stent and side-by-side metal stents as well as inside-type plastic stents, has the potential to broaden the range of drainage options available. Furthermore, in cases in which transpapillary approaches are advantageous, such as when stent placement is particularly challenging, the use of a dual-layer plastic stent across the papilla may offer significant benefits.

## Figures and Tables

**Table 1:** Baseline characteristics of the patients.

	Group R (n = 48)	Group IS (n = 30)	*p* Value
Gender			
Male Female	28	23	0.145
	20	7	
Age, median (range)	73 (44–91)	74.5 (58–86)	0.406
Etiology			
Biliary tract cancer	43	22	0.0338
Liver metastasis	2	7	
HCC	1	1	
Metastasis of lymph node	1	0	
Disseminated nodule	1	0	

**Table 2:** Data regarding the R group and the IS group.

	Group R (n = 48)	Group IS (n = 30)	*p* Value
Previous endoscopic sphincterotomy	48	30	N.S.
Diameter and size of the placed stent			
7 Fr. 7 cm	6	0	N.S.
7 Fr. 9 cm	0	7	
7 Fr. 10 cm	25	0	
7 Fr. 12 cm	37	39	
7 Fr. 15 cm	11	0	
8.5 Fr. 7 cm	2	0	
8.5 Fr. 10 cm	4	0	
8.5 Fr. 12 cm	7	0	
8.5 Fr. 15 cm	7	0	
Number of the stent			
1	8	16	0.00208
2	29	12	
3	11	2	
Acute cholangitis rate	56.7% (17/30)	50% (24/48)	0.644
TIME to RBO, medium (range)	74 (62–113)	118 (54–322)	0.219
Early adverse events	1	4	N.S.
High-amylase	1	3	
Cholangitis	0	1	

RBO: Recurrent Biliary Obstruction, N.S.: Not Significant.

## Data Availability

The original contributions presented in this study are included in the article. Further inquiries can be directed to the corresponding author.

## References

[1] Wiggers J.K., Coelen R.J., Rauws E.A., van Delden O.M., van Eijck C.H., de Jonge J., Porte R.J., Buis C.I., Dejong C.H., Molenaar I.Q. (2015). Preoperative endoscopic versus percutaneous transhepatic biliary drainage in potentially resectable perihilar cholangiocarcinoma (DRAINAGE trial): Design and rationale of a randomized controlled trial. BMC Gastroenterol..

[2] Kawakami H., Kuwatani M., Onodera M., Haba S., Eto K., Ehira N., Yamato H., Kudo T., Tanaka E., Hirano S. (2011). Endoscopic nasobiliary drainage is the most suitable preoperative biliary drainage method in the management of patients with hilar cholangiocarcinoma. J. Gastroenterol..

[3] Nakai Y., Yamamoto R., Matsuyama M., Sakai Y., Takayama Y., Ushio J., Ito Y., Kitamura K., Ryozawa S., Imamura T. (2018). Multicenter study of endoscopic preoperative biliary drainage for malignant hilar biliary obstruction: E-POD hilar study. J. Gastroenterol. Hepatol..

[4] Nagino M., Hirano S., Yoshitomi H., Aoki T., Uesaka K., Unno M., Ebata T., Konishi M., Sano K., Shimada K. (2021). Clinical practice guidelines for the management of biliary tract cancers 2019: The 3rd English edition. J. Hepato-Biliary-Pancreat. Sci..

[5] Jang S., Stevens T., Parsi M.A., Bhatt A., Kichler A., Vargo J.J. (2022). Superiority of Self-Expandable Metallic Stents Over Plastic Stents in Treatment of Malignant Distal Biliary Strictures. Clin. Gastroenterol. Hepatol..

[6] Kwon C.I., Lehman G.A. (2016). Mechanisms of Biliary Plastic Stent Occlusion and Efforts at Prevention. Clin. Endosc..

[7] Kanno Y., Ito K., Nakahara K., Kawaguchi S., Masaki Y., Okuzono T., Kato H., Kuwatani M., Ishii S., Murabayashi T. (2023). Suprapapillary placement of plastic versus metal stents for malignant biliary hilar obstructions: A multicenter, randomized trial. Gastrointest. Endosc..

[8] Okuno M., Iwata K., Iwashita T., Mukai T., Shimojo K., Ohashi Y., Iwasa Y., Senju A., Iwata S., Tezuka R. (2024). Evaluating optimal bilateral biliary stenting in endoscopic reintervention after initial plastic stent dysfunction for unresectable malignant hilar biliary obstruction: Retrospective cross-sectional study. Dig. Endosc..

[9] Yang H., Deng J., Hu Y., Hong J. (2023). Meta-analysis on clinical outcomes of suprapapillary versus transpapillary stent insertion in malignant biliary obstruction. Surg. Endosc..

[10] Ishiwatari H., Hayashi T., Ono M., Sato T., Kato J. (2013). Newly designed plastic stent for endoscopic placement above the sphincter of Oddi in patients with malignant hilar biliary obstruction. Dig. Endosc..

[11] Kanda Y. (2013). Investigation of the freely available easy-to-use software ’EZR’ for medical statistics. Bone Marrow Transpl..

[12] De Souza G.M.V., Ribeiro I.B., Funari M.P., de Moura D.T.H., Scatimburgo M.V.C.V., de Freitas Júnior J.R., Sánchez-Luna S.A., Baracat R., de Moura E.T.H., Bernardo W.M. (2021). Endoscopic retrograde cholangiopancreatography drainage for palliation of malignant hilar biliary obstruction—Stent-in-stent or side-by-side? A systematic review and meta-analysis. World J. Hepatol..

[13] Oh D.Y., Ruth He A., Qin S., Chen L.T., Okusaka T., Vogel A., Kim J.W., Suksombooncharoen T., Ah Lee M., Kitano M. (2022). Durvalumab plus Gemcitabine and Cisplatin in Advanced Biliary Tract Cancer. NEJM Evid..

[14] Kelley R.K., Ueno M., Yoo C., Finn R.S., Furuse J., Ren Z., Yau T., Klümpen H.J., Chan S.L., Ozaka M. (2023). Pembrolizumab in combination with gemcitabine and cisplatin compared with gemcitabine and cisplatin alone for patients with advanced biliary tract cancer (KEYNOTE-966): A randomised, double-blind, placebo-controlled, phase 3 trial. Lancet.

[15] Kurita A., Uza N., Asada M., Yoshimura K., Takemura T., Yazumi S., Kodama Y., Seno H. (2022). Stent placement above the sphincter of Oddi is a useful option for patients with inoperable malignant hilar biliary obstruction. Surg. Endosc..

[16] Kogure H., Kato H., Kawakubo K., Ishiwatari H., Katanuma A., Okabe Y., Ueki T., Ban T., Hanada K., Sugimori K. (2021). A Prospective Multicenter Study of “Inside Stents” for Biliary Stricture: Multicenter Evolving Inside Stent Registry (MEISteR). J. Clin. Med..

[17] Nakamura S., Ishii Y., Serikawa M., Tsuboi T., Kawamura R., Tsushima K., Hirano T., Mori T., Uemura K., Chayama K. (2021). Utility of the inside stent as a preoperative biliary drainage method for patients with malignant perihilar biliary stricture. J. Hepatobiliary Pancreat. Sci..

[18] Ishiwatari H., Kawabata T., Kawashima H., Nakai Y., Miura S., Kato H., Shiomi H., Fujimori N., Ogura T., Inatomi O. (2023). Clinical Outcomes of Inside Stents and Conventional Plastic Stents as Bridge-to-Surgery Options for Malignant Hilar Biliary Obstruction. Dig. Dis. Sci..

[19] Coene P.P., Groen A.K., Cheng J., Out M.M., Tytgat G.N., Huibregtse K. (1990). Clogging of biliary endoprostheses: A new perspective. Gut.

